# Glutathione S-Transferases in Cancer

**DOI:** 10.3390/antiox10050701

**Published:** 2021-04-29

**Authors:** Rahul Raj Singh, Katie M. Reindl

**Affiliations:** Department of Biological Sciences, North Dakota State University, Fargo, ND 58108, USA; rahul.r.singh@ndsu.edu

**Keywords:** Glutathione S-transferases (GSTs), antioxidants, cancer-cell signaling, chemoresistance, xenobiotic compounds, metabolism, GST inhibitors, glutathionylation, oxidative stress, JNK, apoptosis, patient survival

## Abstract

In humans, the glutathione S-transferases (GST) protein family is composed of seven members that present remarkable structural similarity and some degree of overlapping functionalities. GST proteins are crucial antioxidant enzymes that regulate stress-induced signaling pathways. Interestingly, overactive GST proteins are a frequent feature of many human cancers. Recent evidence has revealed that the biology of most GST proteins is complex and multifaceted and that these proteins actively participate in tumorigenic processes such as cell survival, cell proliferation, and drug resistance. Structural and pharmacological studies have identified various GST inhibitors, and these molecules have progressed to clinical trials for the treatment of cancer and other diseases. In this review, we discuss recent findings in GST protein biology and their roles in cancer development, their contribution in chemoresistance, and the development of GST inhibitors for cancer treatment.

## 1. Introduction

Glutathione S-transferases (GSTs) are a multigene family (EC 2.5.1.18) of eight dimeric enzymes that are classified based on their amino acid sequences and substrate specificity as alpha (A), kappa (K), mu (M), omega (O), pi (P), sigma (S), theta (T), and zeta (Z) [1]. Depending on their subcellular location, GSTs are grouped as cytoplasmic (A, P, M, S, T, Z), mitochondrial (K), or membrane-bound (Membrane Associated Proteins in Eicosanoid and Glutathione metabolism) [2]. GSTs are phase-II detoxification enzymes found in most life forms and vital for maintaining cellular homeostasis [3]. GSTs play a cytoprotective role primarily by catalyzing the conjugation reaction of reduced glutathione (GSH) and reactive electrophiles generated by cytochrome P450 metabolism to form GSH conjugates [4]. The resulting GSH conjugates are either excreted via bile or transported to the kidney where: (1) the γ-glutamyl moiety is cleaved by γ-glutamyl transpeptidase; (2) the glycine is cleaved by dipeptidase; and (3) the cysteine is N-acetylated [5].

In addition to their detoxification roles, GSTs are known for their functions in cell signaling, post-translational modification, and resistance to chemotherapeutic agents [6]. For example, the pi and mu classes of GSTs regulate the mitogen-activated protein (MAP) kinase pathway that governs cell survival and cell death signals via direct interactions with c-Jun N-terminal kinase 1 (JNK1) and apoptosis signal-regulating kinase (ASK1) [7]. Additionally, GSTs form complexes with an array of intracellular proteins for their post-translational modification [8]. For instance, protein disulfide isomerase (PDI), peroxiredoxin-VI (Prdx VI), and p53 are common substrates of GST-mediated glutathionylation [9]. Similar to the detoxification process described above, antineoplastic drugs bound to GSH are expelled out of the cells by the membrane-bound GS-X pump, making cancer cells resistant to chemotherapy [10]. Since their discovery in 1961 in rat liver [11], GSTs have gained attention among cancer researchers. The expression of GSTs in all cell types and their abundance in aggressive cancer cells suggest that they play a key role in tumor progression and cancer pathogenicity [12]. Recent developments in the field of redox oncology have shed light on novel functions of GST proteins in cancer cells [13,14]. This review summarizes newly identified functions of GST proteins and their roles in the cellular signaling, metabolism, and survival of cancer cells.

## 2. Structure

**Figure 1:**
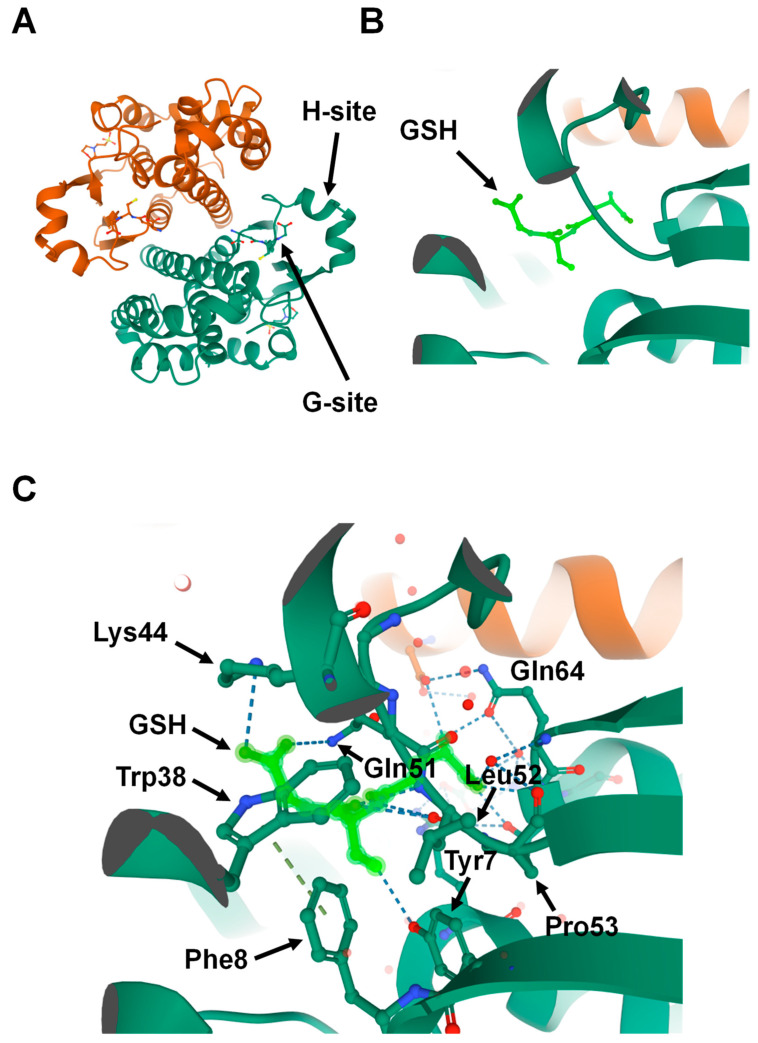
Structure of Glutathione S-transferase Pi 1 (GSTP1) (Protein Data Bank ID: 6GSS). (**A**) Homodimer assembly of GSTP1 showing G- and H-sites. (**B**) Magnified view of the G-site that is occupied by the ligand glutathione (GSH) (shown in light green). (**C**) Glutathione (light green) forms hydrogen-binding interactions with the surrounding amino acids found in the G-site pocket of GSTP1.

Because GSTs are pivotal in drug metabolism, they were among the first cytosolic proteins to be structurally characterized. Porcine GST Pi 1 (GSTP1)was the first member of the family whose structure was determined [15]. Crystallographic studies have revealed that the catalytic GSTs display analogous tertiary structures and exist as homodimers in mammals (Figure 1A) [16]; however, heterodimers of a few cytosolic GSTs have been identified in plants [17]. Currently, no enzymatically active monomers of GST proteins are known [15]. Subsequent structural analyses revealed that all principal GST family members have a basic protein fold consisting of two domains: the N-terminal domain and the C-terminal domain. The GST N-terminal domain fold is similar to other cellular homeostasis and detoxification proteins such as glutathione peroxidases and glutaredoxins. The N-terminal domain constitutes approximately one-third of the protein structure and is made up of a β-α-β-α-β-β-α motif. The β-β-α motif in the N-terminal domain, also known as the G-site, is most conserved among the isoforms and provides the binding site for reduced glutathione (GSH) by recognizing the γ-glutamyl fragment of GSH (Figure 1B).

Interestingly, a proline residue, found at the N-terminal end of strand β3, is conserved among all cytosolic and mitochondrial GSTs. This proline forms hydrogen-bond interactions with the backbone amine group of the GSH-cysteinyl moiety (Figure 1C) [16,18,19]. Global characterization of sequence and structure similarity of GST proteins show two major subgroups: (1) tyrosine-type GSTs (Y-GSTs), which use tyrosine to activate GSH; and (2) S/C-GSTs, which use serine (or cysteine in case of GST Omega (GSTO)) to interact with GSH [20]. However, the C-terminal domain of GSTs constitutes the other two-thirds of the protein structure and is made up of a unique all-α-helical domain [19]. The hydrophobic substrates bind to a cleft between the N- and C-terminal domains known as the H-site. Unlike the G-site, the H-site is highly variable in shape and chemical constitution between classes [21]. This variability in H-site structure determines the substrate selectivity of various GST isozymes [22].

## 3. Metabolism of Xenobiotic Compounds

Exposure to several natural and manufactured substances in the environment accounts for more than two-thirds of all cancer cases in the United States [23]. These environmental factors range from lifestyle choices such as smoking, excessive alcohol consumption, and poor diet to exposure to certain medical drugs, radiation, and environmental chemicals present in the air, water, or food. Environmental carcinogens are known to generate reactive oxygen species (ROS) in the cells [24]. Strong evidence exists suggesting that oxidative stress promotes damage to the cellular components, including proteins, lipids, membranes, and DNA, that play a crucial role in cancer development [25].

Aerobic organisms have a cellular defense system composed of several enzymes that scavenge ROS and protect cells from macromolecular damage [26]. Phase-I detoxification enzymes process the primary steps of xenobiotic detoxification. For these reactions, an array of cytochrome P450 enzymes are utilized, and detoxification is achieved by a series of oxidation-reduction reactions. Due to their electrophilic nature, phase-I metabolites have a high affinity to form adducts with nucleic acids and proteins [27]. However, these cytotoxic intermediate metabolites are readily conjugated to hydrophilic moieties such as reduced glutathione (GSH), glucuronate, and sulfate by phase II enzymes, such as glutathione S-transferases, uridine 5′-diphospho-glucuronosyltransferases (UDP), sulfotransferases, and nicotinamide adenine dinucleotide phosphate-oxidase:quinone oxidoreductase (NQO) [28].

Carcinogens, industrial intermediates, pesticides, and environmental pollutants are the most common substrates for GSTs [29]. Environmental carcinogens such as polycyclic aromatic hydrocarbons (PAHs) that are converted to epoxides via P450 metabolism are pervasive in the modern industrial world and are a threat to general health [30]. PAHs, commonly found in engine exhaust fumes and cigarette smoke, are conventional substrates of GSTs [31]. GSTs primarily carry out the catalytic detoxification of the above-mentioned exogenous compounds via synthesis of mercapturic acids [32]. The γ-glutamyl and the glycine fragment of the resulting glutathione conjugate are trimmed, followed by N-acetylation of the cysteine S-conjugates [33]. It is important to note that GSTs are a part of a unified cellular defense system. They rely on glutathione synthase activity to supply GSH [34] and transporter proteins to export the GSH conjugates [35,36].

The tripeptide, γ-l-glutamyl-l-cysteinyl-glycine, known as glutathione (GSH), is an essential antioxidant in the cell [37]. The synthesis of GSH is a two-step enzymatic reaction mediated by: (1) γ-glutamylcysteine synthetase combining cysteine with glutamate; and (2) glutathione synthetase adding glycine to the dipeptide to produce GSH. The above reactions are coupled with adenosine triphosphate (ATP) hydrolysis [38]. GSH is primarily found in the cytosol with concentrations ranging from 1–3 mM [39]; however, it has also been reported in mitochondria [40] and nucleus [41], where it functions in regulating apoptosis and cell division, respectively. Its primary role is to act as a free-radical scavenger and trap ROS that would otherwise cause irreparable damage to proteins, lipids, and nucleic acids [42]. The significance of the detoxification properties of GSH has been illustrated by depleting its intracellular levels and demonstrating the increased in vitro toxicities of compounds that depend on GSH metabolism, such as chromium [43], cadmium [44], arsenic [45], bleomycin [46], and mitomycin [47]. The detoxification reaction involving GSH is primarily catalyzed by glutathione peroxidases (GPx) through the following reaction

2GSH + H_2_O_2_ → GSSG + 2H_2_O (1)

where hydrogen peroxide (H_2_O_2_) is a low molecular weight, reactive oxygen species. H_2_O_2_ is primarily produced by the superoxide anion via a dismutation reaction [48] and plays critical roles in hypoxia [49], inflammation [50], apoptosis [51], and autophagy [52]. It is important to note that oxidized glutathione (GSSG) is reduced back to GSH by glutathione reductase and at the expense of NADPH through a GSH-restoring system [53] via following reaction

GSSG + NADPH + H^+^ → 2GSH + NADP^+^ (2)

## 4. Cellular Signaling

**Figure 2:**
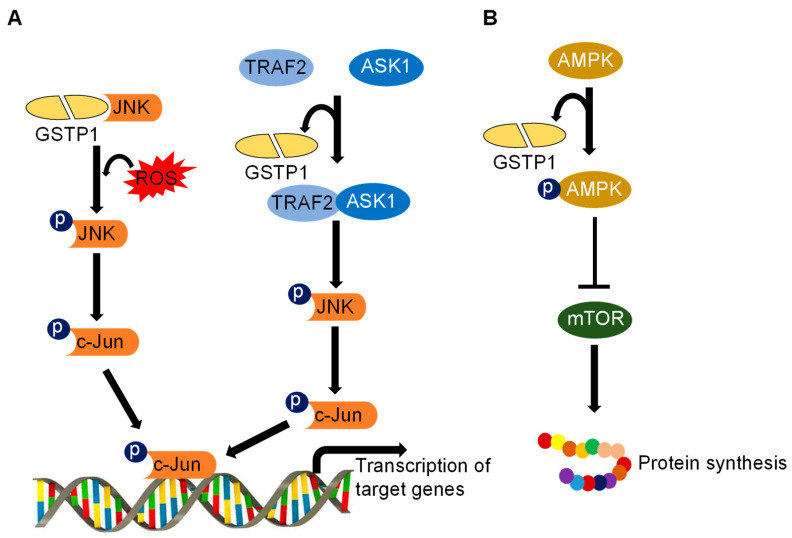
Multifarious roles of GSTP1 in cell signaling. (**A**) Under oxidative stress, the interaction between GSTP1 and c-Jun N-terminal kinase 1 (JNK1) is deterred and leads to phosphorylation of c-Jun and transcription of target genes. Similarly, in the absence of GSTP1, tumor necrosis factor (TNF)-receptor-associated factor 2 (TRAF2), and apoptosis signal-regulating kinase (ASK1) interact and cause phosphorylation of c-Jun. (**B**) Phosphorylation of 5′ adenosine monophosphate-activated protein kinase (AMPK) in GSTP1 knockdown cells inhibits the mechanistic target of rapamycin (mTOR) pathway and impairs protein synthesis.

Besides their glutathione-conjugating activity, GSTs are known to bind structurally distinct non-substrate molecules. Several GST isozymes interact with the members of mitogen-activated protein kinase (MAPK) pathways involved in cell-survival and cell-death signaling mechanisms. This non-enzymatic function of GSTs is achieved by binding to the kinase protein in complex, thus preventing the activation of downstream targets. A specific subtype of the GST protein family, GSTP1, binds to the JNK complex [7,54,55]. Through fluorescence resonance energy-transfer measurements, it was revealed that the C- terminus of the JNK protein is essential for its interaction with GSTP1 [56]. Studies have revealed that dimerization of GSTP1 is critical for enzymatic activity and its interaction with JNK. It was shown that under non-stressed, normal conditions, monomeric GSTP1 binds to JNK and prevents its phosphorylation. However, under oxidative stress conditions, the GSTP1-JNK complex dissociates, allowing phosphorylation of JNK and dimerization of GSTP1 [7,56]. In other words, GSTP1 acts as a sensor of oxidative stress and modulates the JNK signaling pathways for cell survival or apoptosis depending on the level of ROS encountered [56] (Figure 2A). The interaction between GSTP1 and JNK has also been demonstrated in vivo [7]. A higher and constitutive JNK activity was reported in the liver and lungs of transgenic mice in which GSTP1/2 were deleted (GSTP1/2^(−/−)^) compared to the wild-type control. In the same model system, increased DNA-binding activity of AP-1 was reported. In addition to establishing the role of GSTP1 as a JNK inhibitor, this study also demonstrated the role of GSTP1 in regulating the expression of specific downstream targets of the JNK signaling pathway [7].

Additionally, GSTP1 interacts and inhibits the activity of tumor necrosis factor (TNF)-receptor-associated factor 2 (TRAF2), a member of the TNF receptor-associated factor protein family [57]. Human cervical cancer HeLa cells overexpressing GSTP1 suppress TRAF2-induced activation of both JNK and p38. Further, GSTP1 attenuated autophosphorylation of apoptosis signal-regulating kinase 1 (ASK1) and inhibited TRAF2-ASK1-induced apoptosis [58]. On the contrary, silencing of GSTP1 triggered TRAF2-ASK1 association and hyper-activation of ASK1 and JNK [58]. Similar findings have been reported about the interaction of GSTM3 and TRAF6 in cervical cancer cells [13]. Further, GSTP1 knockdown in pancreatic [59] and GSTM knockdown in cervical cancer cells [13] showed reduced phosphorylation of extracellular signal-regulated kinase (ERK1/2),which plays a pivotal role in promoting cell growth and proliferation in many mammalian cell types. We have previously reported that GSTP1 knockdown pancreatic ductal adenocarcinoma (PDAC) cells have impaired growth compared to the control [59]. We hypothesize this phenotype is attributed to the reduced ERK activity upon GSTP1 knockdown. In addition, genetic and pharmacological inactivation of GSTP1 in triple-negative breast cancer showed increased phosphorylation of 5′ adenosine monophosphate-activated protein kinase (AMPK) and acetyl-coenzyme A carboxylase [12]. Phosphorylation and activation of AMPK have previously been demonstrated to reduce growth in breast cancer cells, primarily by inhibiting the mechanistic target of rapamycin (mTOR)-signaling pathway [60] (Figure 2B). Interestingly, GSTP1-knockdown mediated growth inhibition in these cells can be partly rescued by the treatment of AMP kinase inhibitor, dorsomorphin [12].

Other GST isozymes, such as GSTA1, are also known to negatively regulate the mTOR-signaling pathway. Liu et al. showed that overexpression of GSTA1 in hepatocellular carcinoma cells can increase AMPK activity and inhibit the mTOR pathway [61]. They found that hepatocellular carcinoma patients with higher GSTA1 had better prognoses, and GSTA1 overexpression can impair liver cancer cell proliferation and metastasis. Further, in human neuroblastoma cells, Saisawang et al. demonstrated that GSTO1 modulates protein kinase B and MAPK1/2 activation [62]. Using the GSTO1-specific inhibitor, ML175, they showed that GSTO1 enzyme activity inhibits the activation of these kinases and indirectly regulates the survival, growth, and metabolism of neuroblastoma cells.

## 5. Cellular Metabolism

Cancer is often referred to as a metabolic disease, and aberrant metabolism is known to drive the pathogenicity of various neoplasms [63,64]. Escalated aerobic glycolysis and lipid biosynthesis are key in generating cancer cell biomass and regulating signaling mechanisms [65]. Using a reactivity-based chemoproteomic platform, GSTP1 was identified as a chief player that controls cancer cell metabolism in triple-negative breast cancer cells [12]. Genetic or pharmacological inactivation of GSTP1 resulted in reduced lactic acid, ATP, nucleotides, and increased acylcarnitines and lysophospholipids. Upon mapping to metabolic pathways, it was found that inactivation of GSTP1 leads to impaired glycolytic metabolism resulting in reduced ATP as well as reductions in the levels of macromolecular building blocks. It was concluded that GSTP1 interacts with glyceraldehyde-3-phosphate dehydrogenase (GAPDH) and increases its enzyme activity in breast cancer cells [12]. Recently, Hildebrandt et al. [66] and Moellering et al. [67] showed that GAPDH-mediated conversion of 3-phosphoglycerate to 1,3-bisphosphoglycerate is a rate-limiting glycolytic reaction in cancer cells that have heightened aerobic glycolysis. These studies validate that GSTP1-arbitrated GAPDH activation is a critical metabolic hub and, therefore, GSTP1 inhibitors are promising therapeutics for breast cancer patients.

Additionally, tetra-hydroxynonenal (4-HNE), a common byproduct of lipid peroxidation, was found to be reduced in GSTP1-positive prostate cancer patients compared to GSTP1-negative patients, indicating that GSTP1 protects lipids from oxidative damage [68]. The reactive 4-HNE adducts are mutagenic and are often accumulated in various pathological conditions [69,70]. Further research is needed to identify and comprehensively characterize additional metabolic changes that are influenced by GST activity.

## 6. Chemoresistance

Ambiguous early symptoms and the lack of early diagnostic tools for many cancers results in a late diagnosis for many patients [71]. While surgery is the preferred line of treatment, only a small fraction of patients are eligible for resection surgery, especially for pancreatic cancer [72], non-small cell lung carcinoma [73], and glioblastoma [74]. Chemotherapy is used for patients with advanced and metastatic disease [75]. However, chemotherapy has largely been ineffective in many cancers, such as pancreatic ductal adenocarcinoma [76,77]. The primary reason for the dismal performance of various chemotherapies is the development of intrinsic or extrinsic resistance to antineoplastic reagents [78]. Several cellular signaling pathways are known to play critical roles in the development of chemoresistance. Pathways commonly associated with cell growth [79,80], proliferation [81,82], and detoxification [83] have a direct impact on drug efficacy in cancer cells.

Recent evidence supports that enzymes involved in maintaining cellular redox homeostasis, such as GSTs, can detoxify chemotherapeutic drugs [84]. For example, GSTP1’s role in chemoresistance is well established in human ovarian cancer. In Chinese ovarian cancer patients, positive correlations have been reported between the overexpression of GSTP1 and chemoresistance [85]. Interestingly, the response rate to chemotherapy for GSTP1-positive ovarian tumors was significantly lower than the GSTP1-negative tumors in a different cohort [86]. Similarly, an independent epidemiological study identified a drug-resistant phenotype in GSTP1-expressing ovarian tumors in Japanese women [87]. In this cohort, out of the eleven GSTP1-positive samples, ten were drug-resistant, and out of the seventeen GSTP1-negative samples, six showed the drug-resistant phenotype. Further, in the same group, the GSTP1-positive ovarian cancer patients showed shorter survival post-diagnosis than the GSTP1-negative cohort. They concluded that GSTP1 expression in tumor cells is related to drug resistance of patients with epithelial ovarian cancer. Besides, GSTP1 knockdown ovarian cancer cells showed heightened sensitivity (IC_50_) to cisplatin and carboplatin by 2.3- and 4.8-fold, respectively. They reported that cell cycle progression was unaffected; however, cell invasion and migration were significantly reduced in GSTP1 knockdown cancer cells.

In addition to ovarian cancer, GSTP1 is involved in the chemoresistance of other cancer types. Proteomics analysis revealed that GSTP1 is overexpressed in cisplatin- and irinotecan-resistant glioma [88,89], fluorouracil (5-FU)- and cisplatin-resistant gastric cancer cells [90], doxorubicin-resistant prostate cancer cells [91], and adriamycin-resistant breast cancer cells [92]. Further, Yang et al. demonstrated that small RNA-mediated knockdown of GSTP1 significantly increased the apoptosis and DNA damage in adriamycin-treated breast cancer cells [93]. Additionally, breast cancer cells were rescued from apoptosis by overexpressing GSTP1. GSTP1 was found to be upregulated in CLDN6-overexpressing and multidrug-resistant estrogen-receptor positive (ER+) breast cancer cells. Knockdown of CLDN6 reduced the expression and the enzyme activity of GSTP1 and increased the cytotoxicity of adriamycin, 5-FU, and cisplatin in ER+ breast cancer cells. Similar observations were made in triple-negative breast cancer cells [93]. Complementing the observations mentioned above, Ogino et al. found that the subcutaneous tumors generated from GSTP1 knockdown esophageal squamous cancer cells treated with cisplatin showed an impressive reduction in size compared to the GSTP1 knockdown and cisplatin treatment group alone [94]. Li et al. made similar observations where they showed GSTP1 inhibition sensitizes lung cancer stem cells to cisplatin treatment [95]. Small RNA-mediated knockdown of GSTP1 in lung cancer cells showed increased activation of JNK and increased camptothecin-induced apoptosis [96]. Camptothecin is a naturally occurring alkaloid that is known for its antineoplastic activity because of its ability to target DNA topoisomerase I specifically. These emerging pieces of evidence suggest that the efficacy of chemotherapy and the overall outcome in cancer patients could be significantly improved if used in combination with GSTP1 inhibitors.

Tumor relapse has been linked to a small number of therapy-resistant cells known as cancer stem cells that survive treatment or develop during post-therapeutic remission [97]. Growing evidence suggests that cancer stem cells are responsible for tumor initiation [98], progression [99], metastasis [100], and drug resistance [101,102,103]. Higher protein levels of GST isoforms have been reported in cancer stem cells, which is a primary reason for their drug-resistant phenotype. Tanaka et al. observed that knockdown of GSTP1 in colorectal cancer stem cells significantly reduced tumor growth [104]. Further, increased chemoresistance of stem-like non-small cell lung cancer cells is also linked to increased protein expression of GSTP1 [105]. Abundant levels of GST isozymes in cancer and cancer stem cells are correlated to the multidrug-resistant phenotype [106,107]; however, most antineoplastic agents are poor substrates for GST isozymes [108]. Thus, the GST-mediated drug resistance could be explained by alternative roles of GSTs other than detoxification of chemotherapeutic drugs [6]. These studies have established the role of GSTP1 in the chemoresistance of anatomically diverse cancer cells. This has led to an increased focus on targeting the GST isozymes to increase the efficacy of chemotherapeutic drugs and improve patient survival [109,110,111]. The role of GSTP1 in resistance to chemotherapy and the respective cancer model is summarized in Table 1. 

Antineoplastic agents such as cisplatin [112,113], cytarabine [114], and gemcitabine [115,116] induce cell death via JNK and p38MAPK pathways. Given that GSTP1 is a direct inhibitor of JNK activity, it is speculated that GSTP1 overexpression is associated with many drug-resistant tumors [90,91,92]. Elevated levels of GSTP1 are shown in pancreatic [59] and triple-negative breast cancer cells [12], where it interferes with the cellular signaling processes that influence cell survival, proliferation, and apoptosis. These non-enzymatic functions of GSTP1 provide an explanation for drug-resistance in GSTP1-overexpressing tumors to chemotherapeutic agents that are poor substrates for this enzyme [108].

## 7. GSTs Glutathionylate Various Proteins

S-glutathionylation occurs through the reversible addition of a glutathione donor to the thiolate moiety of cysteines in target proteins [117]. Like other post-translational modifications, glutathionylation can change the charge, mass, structure, and function of fully translated proteins [118]. Glutathionylation occurs primarily through a thiol-disulfide exchange reaction, as shown below, where ProSH represents the protein with a free cysteine residue [119].

ProSH + GSSG → ProSSG + GSH(3)

Glutathionylation is also known to occur via direct oxidation of a target protein as represented below:

GSH + ProSH → ProSSG(4)

The GST-protein family members are known to post-translationally modify several target proteins by catalyzing the forward S-glutathionylation reaction [9]. The earliest evidence of their role in post-translational modification comes from GSTP1 knockout mice. Zhi-Wei et al. showed that GSTP1 knockout mice had reduced global protein glutathionylation levels compared to wild-type animals [120]. Additionally, they reported that cells expressing mutated GSTP1 polymorphic forms and lacking the catalytically active tyrosine residue had reduced glutathionylation activity.

Peroxiredoxins (Prxs), a family of thiol-specific peroxidase enzymes, are known targets for GSTP1-mediated reversible glutathionylation [121]. Ubiquitously expressed Prxs are found in all kingdoms and are located in all cellular components [122]. These enzymes perform their antioxidant function by reducing H_2_O_2_ and organic peroxides utilizing the intracellular thiols [123]. However, the catalytic cysteine in Prx enzymes is susceptible to oxidation and subsequent loss of peroxidase activity. GSTP1 facilitates the glutathionylation of the previously oxidized cysteine residue, thereby restoring the peroxidase activity [124]. The two major subclasses of Prxs, 1-cys Prx (also known as Prdx VI) and 2-cys Prx, are substrates for glutathionylation [121,125]. Prdx VI, a multi-tasking antioxidant enzyme, is the only mammalian peroxiredoxin that can reduce phospholipid hydroperoxides through its glutathione peroxidase activity [126]. The catalytically active cys-47 residue is buried in the hydrophobic core of the Prdx VI protein. Following peroxide reduction, the oxidized cys-47 is accessed by GSH-loaded GSTP1 to reactivate Prdx VI [127]. Persuasive evidence suggests that different polymorphic forms of GSTP1 can differentially mediate Prdx VI activation and thereby affect the response to ROS/reactive nitrogen species (RNS). For instance, GSTP1-1A, the most abundant polymorphic form of GSTP1, has a higher affinity for Prdx VI compared to GSTP1-1B or 1D [128]. Moreover, breast cancer cells transiently transfected with GSTP1-1A showed significantly higher peroxidase activity than those transfected with GSTP1-1B [128]. The differences in the activity can be attributed to the variation in the relative distance between oxidized cys-47 and the activated GSH bound to the GSTP1 molecule in the different polymorphic forms.

GST-mediated glutathionylation affects the function of additional proteins such as nitric oxide synthase (NOS). NOS contains highly conserved cys-689 and cys-908 residues that are susceptible to S-glutathionylation [129]. NOS activity is reduced upon glutathionylation, resulting in lower nitric oxide (NO) levels and impaired endothelium-dependent vasodilation [129]. Further, using in vivo hypertensive-rat models, it was validated that endothelial-NOS glutathionylation increases with oxidative stress and has direct implications in vascular dysfunction [129].

Post-translational modification and folding of secretory and transmembrane proteins occur in the endoplasmic reticulum (ER). However, if the influx of nascent, unfolded polypeptides outpaces the folding capacity of ER, the homeostasis of ER is impaired resulting in unfolded protein response (UPR) [130]. Studies have shown that UPR is an underlying cellular mechanism of various human diseases such as ischemia, Friedreich’s ataxia, Alzheimer’s disease, type 2 diabetes, and cystic fibrosis [131,132,133]. Evidence increasingly shows interrelations between S-glutathionylation and UPR [134]. Protein disulfide isomerase (PDI) is a principal component of the cellular protein folding machinery [135]. Utilizing mass spectrometry and circular dichroism, Townsend et al. showed that S-glutathionylation decreases the isomerase activity of PDI and is potentially an upstream signaling event in UPR [134,136].

Exposed thiol groups determine the supramolecular structure of cytoskeletal proteins [137]. It has been shown that glutathionylation of these sites can affect protein function by protecting them from irreversible oxidation [138] or inhibiting polymerization [139]. Actin has been identified as a common substrate for glutathionylation in endothelial [140] and gastric mucosal cells [138]. In response to oxidative stress, Dalle-Donne et al. reported that S-glutathionylation of cys-374 impairs the rate of actin polymerization [139]. The authors added that a reduced rate of actin polymerization was partially due to the slow addition of actin monomers to the growing actin filament chain. S-glutathionylation of actin deregulates the soluble:filamentous protein ratios and affects various cytoskeletal functions. In the ischemia-reperfusion injury rat model, Chen et al. described that glutathionylated actin affects cell adhesion and has a weaker affinity for tropomyosin [141]. Additional cytoskeletal proteins are targets of glutathionylation. Rapid and reversible glutathionylation of beta-tubulin was observed in oxidation-stressed human endothelial cells [140]. In addition, using two-dimensional electrophoresis and mass spectrometry fingerprinting, Fratelli et al. reported that cytoskeletal proteins such as vimentin, cofilin, myosin, and profilin are glutathionylated in human T-cell blasts exposed to oxidative stress [8]. Further, using atomic force microscopy, electron microscopy, and hydrogen-deuterium exchange mass spectrometry, Kaus-Drobek et al. demonstrated that glutathionylation of cys-328 of human vimentin inhibits filament elongation [142]. In brief, S-glutathionylation of cytoskeletal proteins disrupts their polymerization and contributes to anti-metastatic activity in cancer cells.

p53, a principal transcription factor, plays a pivotal role in DNA repair, cell cycle control, differentiation, and tumor suppression through transcriptional activation of an array of target genes in response to a variety of endogenous and exogenous stimuli [143]. Functional inactivation of p53 gives rise to various unstable genomes that cause more than 60% of all human cancers [144]. Human p53 encodes ten cysteine residues that reside in its DNA binding domain [145]. Velu et al. reported that p53 is a substrate for glutathionylation, and this post-translational modification reorganized p53′s structure and reduced its affinity for DNA binding [146]. Interestingly, Yusuf et al. observed that oxidative stress and DNA damage treatments increased the levels of glutathionylated p53 [147]. Moreover, using immunohistochemistry, they reported abundant levels of glutathionylated p53 in human prostate adenocarcinoma and melanoma tissues. Overall, glutathionylation of p53 might be a physiologically relevant phenomenon that occurs as a cellular defense mechanism in response to stress stimuli. This modification is known to revamp gene expression for cancer cell survival [148]. Further, increased GST activity in tumor cells could promote oncogenesis through glutathionylation and inhibition of key proteins such as p53.

Protein kinase C (PKC) isozymes are major cellular signaling molecules that play important roles in proliferation, invasion, tumorigenesis, and metastasis [149]. The earliest evidence suggesting the role of PKC in tumor progression was its identification as a cellular receptor for phorbol esters [150]. Since then, studies have shown that the overexpression of PKC drives tumor development via synergistic activation of several cell-survival and mitotic pathways, including nuclear factor-κB (NF-κB), signal transducer and activator of transcription 3 (Stat3), phosphatidylinositol 3-kinase/protein kinase B (PI3K/Akt), and extracellular signal-regulated kinase (ERK) [151,152]. Ward et al. showed that PKC isolated from the rat brain is inactivated by glutathionylation in the presence of diamide and glutathione [153]. Importantly, they concluded that the antagonistic role of GSH in tumor progression is mediated via oxidative inactivation of PKC isozymes.

Glutathionylation is additionally known to inhibit the activity of various enzymes involved in energy metabolism. Complex I [154], cytochrome oxidase [8], ATPase [14], carbonic anhydrase [155], and pyruvate dehydrogenase [156] are known targets of glutathionylation. Glutathionylation inhibits the activity of these metabolic enzymes. These, and additional proteins that are post-translationally modified by glutathionylation, are summarized in Table 2.

## 8. GST Inhibitors and Their Therapeutic Implications

Considering the roles of GST proteins in promoting tumor pathogenicity and chemoresistance, attempts have been made to develop specific GST inhibitors to reduce tumor growth and enhance the cytotoxic properties of chemotherapeutic drugs [109,110,175]. GST inhibitors are classified based on their binding activity and structure. Molecules that can bind to the G- or H-site of GST proteins, glutathione peptidomimetics, and several natural compounds have been identified as GST inhibitors. Some of the commonly studied GST inhibitors are discussed below.

### 8.1. Inhibitors That Bind to the G-Site

**Figure 3:**
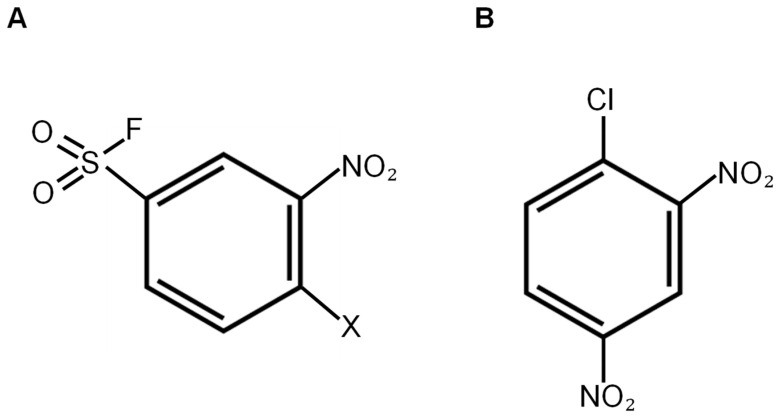
Structure of inhibitors that bind to the G-site of GST proteins. (**A**) Covalent inhibitor benzene sulfonyl fluoride (BSF)-type ligand (X=Cl, F) and (**B**) 1-chloro-2,4-dinitrobenzene (CDNB).

Crystallographical studies have found that different GST isoforms have unique G-site structures [176]. This information was instrumental in developing inhibitors for specific GST subtypes. Interestingly, the G-site accepts only glutathione as a substrate, and as a result, glutathione is used as a prototype to develop G-site inhibitors. However, the high intracellular concentration of glutathione presents the biggest challenge for the development of competitive G-site inhibitors for GST proteins. Because the Tyr7 in the active site of GSTP1 extracts the thiol proton of glutathione, Shishido et al. designed GSTP1 inhibitors by placing the electrophilic reactive group around the thiol group of GSH [177]. CD spectral analysis revealed no structural modifications in the presence of the inhibitor, validating no evidence of protein denaturation. Liquid Chromatography with tandem mass spectrometry (LC-MS/MS) confirmed the covalent binding of the inhibitor to GSTP1. To circumvent using high concentrations of the inhibitors, cell membrane permeable benzene sulfonyl fluoride (BSF)-type covalent inhibitors (Figure 3A) were developed by the same research group [178]. BSF-type covalent GST inhibitors used 1-chloro-2,4-dinitrobenzene (CDNB), a major substrate of GST proteins, as a structural backbone (Figure 3B).

The irreversible binding of the BSF-type inhibitors was analyzed by washout assays, and inhibition of GST enzymatic activity upon treatment by these compounds was analyzed in human non-small-cell lung adenocarcinoma cells. Interestingly, covalent inhibitors showed prolonged inactivation of GST enzymes and hold promise for use as antineoplastic agents against GSTP1-overexpressing tumors. Additionally, amitriptyline, a commonly prescribed drug for clinical depression, significantly inhibits the activity of GSTP1 and GSTA1 by binding to their G-sites; however, the binding of amitriptyline to the GST proteins is reversible [179].

**Figure 4:**
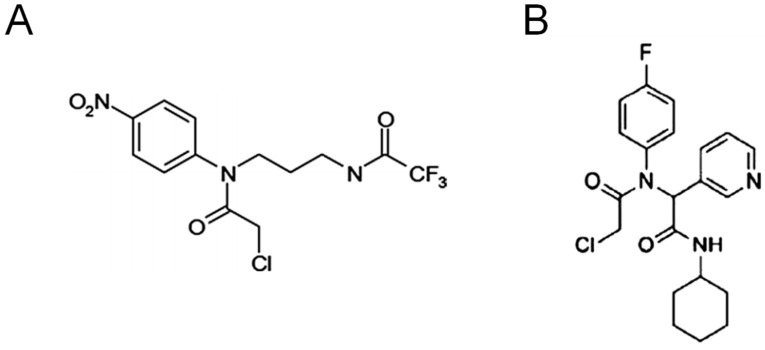
Structure of covalent inhibitors that specifically bind to the active site of Glutathione S-transferase Omega-1 (GSTO1) protein. (**A**) ML175 and (**B**) KT53.

Using fluorescent-activity-based probes, Bachovchin et al. reported the identification of α-chloroacetamide compounds as specific GSTO1 inhibitors [180]. These compounds, specifically ML175 (Figure 4A) and KT53 (Figure 4B), react irreversibly with the cysteine in the active site of GSTO1. Interestingly, Tsuboi et al. demonstrated that the co-treatment of KT53 and cisplatin significantly decreased cell survival compared to KT53 and cisplatin alone [181]. Similar findings have been reported in cisplatin-resistant ovarian cancer cell lines [182,183].

### 8.2. Inhibitors That Bind to the H-Site

**Figure 5:**
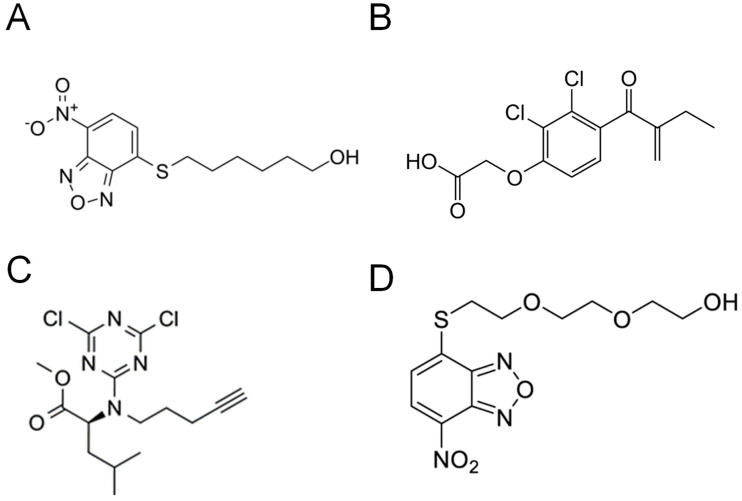
Structure of inhibitors that bind to the G-site of GST proteins. (**A**) 6-(7-nitro-2,1,3-benzoxadiazol-4-ylthio)hexanol (NBDHEX), (**B**) ethacrynic acid, (**C**) LAS17, and (**D**) MC3181 (2-(2-(2-((7-nitrobenzo[c][1,2,5]oxadiazol-4-yl)thio)ethoxy)ethoxy)ethanol), an NBDHEX analogue.

Several compounds can bind to the H-site of GST proteins and inhibit the enzymatic activity of the same. Since the H-site can be occupied by a variety of substrates, it is particularly difficult to develop specific inhibitors for GST subtypes targeting the H-site. Recently, a potent inhibitor of GSTs, 6-(7-nitro-2,1,3-benzoxadiazol-4-ylthio)hexanol (NBDHEX) (Figure 5A), was identified that showed anti-proliferative properties in various cancer cells [184,185]. Structural analysis of GSTP1 and GSTM1 bound to NBDHEX showed that it binds to these subtypes in a similar manner by interacting with aromatic side chains (Tyr108 of GSTP1 and Tyr115 of GSTM2) [186]. The inhibition of GST activity by NBDHEX was demonstrated by the release of GSTP1 from the GSTP1-JNK and GSTP1-TRAF2 complexes upon NBDHEX treatment [57,187]. Further, increased caspase-dependent apoptosis was reported in NBDHEX treated cancer cells [3], including MDR1-expressing leukemia cells [185]. It was concluded that doxorubicin-resistant cancer cells are susceptible to drug treatment if treated with GSTP1 inhibitors such as NBDHEX. An additional H-site binder, ethacrynic acid (Figure 5B), has also been investigated for its GSTP1-inhibitory properties [188]. Li et al. reported significant cytotoxic effects of ethacrynic acid derivatives in human leukemia cells [189].

Furthermore, Crawford et al. synthesized a library of twenty dichlorotriazine probes via tosylating 4-pent-yn-1-ol [190]. Of the twenty compounds investigated, LAS17 (Figure 5C) was reported to specifically inhibit GSTP1 activity by covalent modifications. Moreover, Louie et al. showed that LAS17 treatment of triple-negative breast cancer cells impaired GSTP1 activity and reduced cell growth and proliferation [12]. Further, LAS17 treatment of the immunodeficient mice bearing xenograft breast tumors showed an impressive reduction in tumor weight and volume compared to the untreated controls.

### 8.3. Glutathione Peptidomimetics

**Figure 6:**
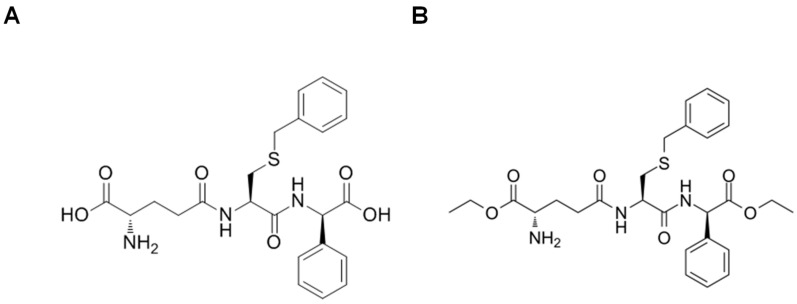
Structure of GSH peptidomimetics (**A**) TLK117 and (**B**) Ezatiostat (TLK199).

γ-glutamyl-S-(benzyl)cysteinyl-R-(−)-phenyl glycine diethyl ester, commonly known as TER199, is the well-studied peptidomimetic analog of glutathione. O’Brien et al. demonstrated that TER199-treated mouse fibroblast cells showed increased expression of the multidrug resistance-associated protein, MRP1, and γ-glutamyl cysteine synthetase. They concluded that GSTP1 inhibition could increase the efficacy of chemotherapeutic drugs and glutathione biosynthesis [191]. Another glutathione peptidomimetic, TLK117 (Figure 6A), has been studied intensively in the context of lung fibrosis. McMillan et al. demonstrated that TLK117- mediated inhibition of GSTP1 blocked lung fibrogenesis through JNK signaling [192]. Ezatiostat, or TLK199 (Figure 6B), is a glutathione analog and a commonly used GSTP1 inhibitor. TLK199 treatment of mouse fibroblast cells showed disrupted GSTP1 binding to JNK and ERK2 [193]. Currently, TLK199 is commercially sold as Telintra^®^ to treat myelodysplastic syndrome patients [193,194].

### 8.4. Natural Compounds

**Figure 7:**
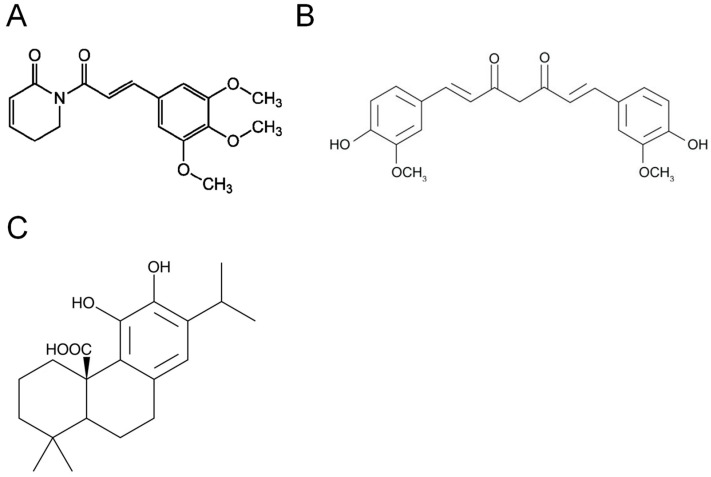
Structure of natural compounds that bind to the GST proteins and inhibit their activity. (**A**) Piperlongumine is a bioactive alkaloid obtained from *Piper longum*, (**B**) Curcumin is an antioxidant obtained from *Curcuma longa*, and (**C**) Carnosic acid is an antioxidant and an anti-inflammatory agent obtained from *Rosmarinus officinalis*.

Chemotherapy is the primary treatment for patients with early and advanced stages of cancer. However, it is common for patients to respond poorly to conventional antineoplastic drugs. Lately, several independent studies have shown that various dietary agents, commonly used in Asian cuisine, show protective effects against multiple diseases, including cancer [195,196,197]. Interestingly, many of these compounds investigated target the cellular antioxidant system. For instance, a bioactive alkaloid compound obtained from *Piper longum*, piperlongumine (Figure 7A), is selectively toxic to cancer cells [198]. Structural and biochemical analyses using x-ray crystallography revealed that piperlongumine, upon entering a cell, hydrolyzes to hydroxypiperlongumine and binds to GSTP1 as a glutathione conjugate [199]. Hydroxypiperlongumine sits deeper into the H-site of GSTP1 that allows four hydrogen-bonding interactions overall. This unique positioning additionally mediates the formation of van der Waals interactions between the side chain of Ile104 and the aliphatic backbone of hydroxypiperlongumine. Piperlongumine treatment causes oxidative stress by inhibiting the activity of GSTP1 and elevating the ROS levels [200]. Hang et al. showed piperlongumine treatment in head and neck cancer-xenograft mouse model reduced tumor growth and increased oxidative stress [201]. Similar results were found in a pancreatic orthotopic tumor mouse model [200].

Additionally, curcumin (Figure 7B), a natural compound extracted from *Curcuma longa*, has antioxidant and chemopreventive properties [202,203]. Duvoix et al. demonstrated that the previously observed activation of NF-κB and anticancer properties in curcumin-treated cells is because of the inactivation of GSTP1 [204]. Carnosic acid (Figure 7C), a phenolic diterpene is extracted from *Rosmarinus officinalis* and is known for its antioxidant and anti-inflammatory properties [202]. Ceylan et al. demonstrated that carnosic acid acts as a competitive inhibitor of GSTO1 [203].

Drug discovery and development have identified GST inhibitors as promising therapeutic targets to counter drug resistance in cancer patients. Convincing data suggest inhibiting GSTP1 protein levels and activity can increase oxidative stress, impair cancer-cell survival, reduce chemoresistance, and improve overall survival in patients [12,13,59]. However, one of the significant roadblocks that GST inhibitors encounter in clinical trials is their insufficient specificity. For example, it was reported that NBDHEX could impair enzyme activity of all GST enzymes, and exhibits a higher affinity to GSTM2 than any other GST isoform [186]. Therefore, to specifically inhibit GSTP1 and treat drug resistance, novel NDBHEX analogues have been developed and tested in human melanoma [205,206]. Among them, 2-(2-(2-((7-nitrobenzo[c][1,2,5]oxadiazol-4-yl)thio)ethoxy)ethoxy)ethanol, also known as MC3181, (Figure 5D) significantly reduced cancer growth and metastasis in vemurafenib-resistant melanoma [207]. However, little is known about the efficacy of these analogues as stand-alone or combination therapeutics. Additionally, the toxicity of GST inhibitors in normal cells is not thoroughly investigated. Therefore, efforts are needed to identify and evaluate novel GST inhibitors that are isoform-specific, overcome drug resistance, and improve overall patient survival.

## 9. Prognostic Impact of GST Protein Expression

**Figure 8:**
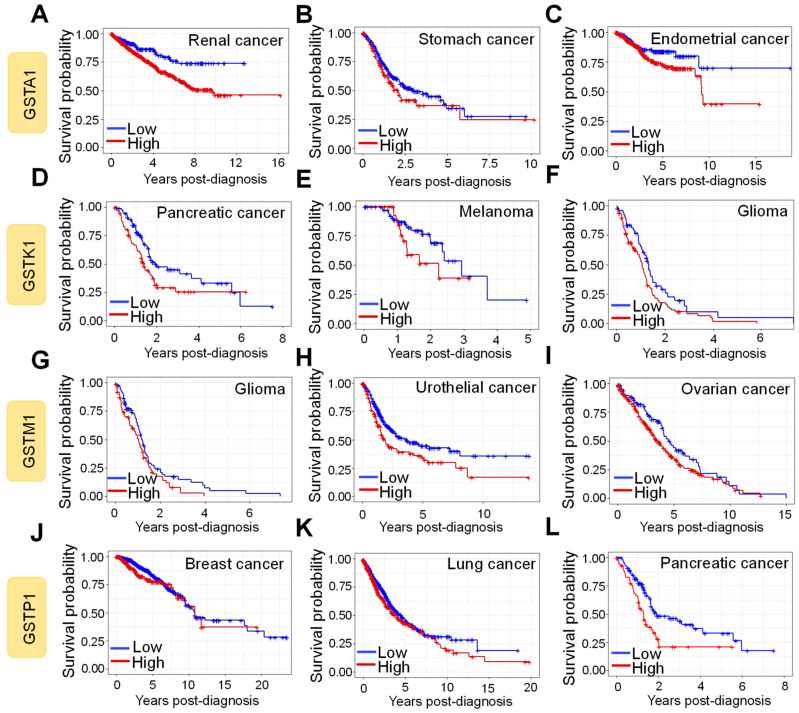
Expression of GST proteins is negatively correlated with patient survival for some human cancers. The Human Protein Atlas was mined for Glutathione S-transferase Alpha 1 (GSTA1) (**A**–**C**), Glutathione S-transferase Kappa 1 (GSTK1) (**D**–**F**), Glutathione S-transferase Mu 1 (GSTM1) (**G**–**I**), and Glutathione S-transferase Pi 1 (GSTP1) (**J**–**L**) mRNA expression in cancer patients relative to their corresponding years of survival post-diagnosis. The patients were divided in high- (red) and low- (blue) GST expressing groups. The Kaplan–Meier survival plots were constructed in RStudio.

Gene polymorphisms within the GST family of proteins are commonly reported in the human population [208]. Evidence suggests that the polymeric forms of GST proteins, most often arising from single-nucleotide polymorphisms (SNPs), have altered enzyme activity. This influences the detoxification of carcinogenic compounds, leads to the accumulation of DNA damage, and by implication, increases the risk of cancer development. Although GST proteins are overexpressed in many tumor tissues, the analysis of the impact of their overexpression on survival has generated differing results. To investigate the survival outcome of GST overexpression in cancer patients, we retrieved the publicly available fragments per kilobase of transcript per million mapped reads (FPKM) values for GST genes and the respective patient survival probability from The Human Protein Atlas [209]. The correlation between expression level and patient survival was examined by plotting the Kaplan-Meier survival plots. We determined that the overexpression of GSTA1 is negatively correlated with patient survival post-diagnosis for renal (*n* = 877) (Figure 8A), stomach (*n* = 354) (Figure 8B), and endometrial (*n* = 541) (Figure 8C) cancer. Similarly, a negative correlation was identified between overexpression of GSTK1 and patient survival for pancreatic cancer (*n* = 176) (Figure 8D), melanoma (*n* = 102) (Figure 8E), and glioma (*n* = 153) (Figure 8F) patients.

Further, identical correlations were found for GSTM1 and GSTP1 for glioma (*n* = 153) (Figure 8G), urothelial cancer (*n* = 406) (Figure 8H), ovarian cancer (*n* = 373) (Figure 8I), breast cancer (*n* = 1075) (Figure 8J) [210], lung cancer (*n* = 994) (Figure 8K), and pancreatic cancer (*n* = 176) (Figure 8L) [59] patients. However, poor patient survival with the overexpression of GST proteins is not uniformly corroborated. For example, high GSTP1 expression improved overall survival in epithelial ovarian cancer [211] and maxillary sinus squamous cell carcinoma patients from China [212]. The contrasting reports can be attributed to the patient population, polymeric forms of GST proteins [213], and treatment regime variations among the studies. Despite the lack of clarity on the impact of overactive GST proteins on the overall survival of cancer patients, there is a popular consensus that higher expression of GST proteins drives tumor pathogenicity and results in poor outcomes [12,13,59,214]. The conflicting data currently makes GST proteins an unreliable prognostic marker for cancer-patient survival.

## 10. Conclusions and Future Directions

GST proteins have complex biology and play multifaceted roles in cancer cells. These enzymes are a crucial component of the cellular antioxidant system and play critical roles in maintaining cellular homeostasis. Under normal physiological conditions, GSTP1 can glutathionylate multiple proteins, including various transcription factors and oncogenes. Conversely, under oxidative stress, GSTP1 can trigger MAPK- and caspase-mediated apoptotic signaling pathways. Interestingly, recent findings suggest that GST enzymes play important roles in cancer development and chemoresistance. However, kinetic and functional studies have revealed that most antineoplastic agents are poor substrates of GSTs with a weaker catalytic constant for the conjugation reaction. Therefore, researchers have shifted their focus to investigating the role of GSTs in various cellular functions, such as regulating kinases and the post-translational processes of diverse proteins.

Multiple studies have demonstrated that GST proteins are overexpressed in many human cancers. Their overexpression contributes to poor outcomes and is negatively correlated with patient survival. However, GSTP1 is not considered a diagnostic marker in clinical practices. We suggest that GSTP1, along with a combination of other biomarkers, may identify a high-risk population that is susceptible to developing cancer. In conclusion, recent studies have established the role of GSTP1 and other GST isozymes in cancer development, progression, metastasis, and resistance to antineoplastic drugs. Active research in the field of antioxidants and redox biology has narrowed to GSTP1 as a promising therapeutic target for cancer treatment. GSTP1 inhibitors can potentially be used in the future to enhance the efficacy of chemotherapy and overcoming drug resistance. However, to use these inhibitors safely for cancer treatment, research is needed to characterize their impact on normal cells and the long-term effects.

## Figures and Tables

**Table 1:** The role of glutathione S-transferases (GST) proteins in the chemoresistance of different cancer model systems is summarized below.

Tumor Model	Anti-Neoplastic Agent	Outcome	Reference
Ovarian cancer	Cisplatin, doxorubicin	Response rate lower in GSTP1-positive patients	[85,86,87]
Glioma	Cisplatin, irinotecan	GSTP1 is overexpressed in resistant tumors	[88,89]
Gastric cancer	Fluorouracil (5-FU), cisplatin	GSTP1 is overexpressed in resistant tumors	[90]
Prostate cancer	Doxorubicin	GSTP1 is overexpressed in resistant tumors	[91]
Breast cancer	Adriamycin	GSTP1 is overexpressed in resistant tumors	[92]
Breast cancer	Adriamycin	Increased apoptosis and DNA damage upon GSTP1 knockdown	[93]
Esophageal squamous cancer cells	Cisplatin	Synergistic effect of GSTP1 knockdown and cisplatin treatment	[94]
Lung cancer stem cells	Cisplatin	Synergistic effect of GSTP1 inhibition and cisplatin treatment	[95]
Lung cancer	Camptothecin	Increased apoptosis upon GSTP1 knockdown and camptothecin treatment	[96]
Lung cancer stem cells	Cisplatin	miRNA-mediated inhibition of GSTP1 reverses cisplatin resistance	[105]

**Table 2:** Proteins that are susceptible to glutathionylation and the resulting effects on their activity are summarized below. Prx, peroxiredoxins; NOS, nitric oxide synthase; PDI, protein disulfide isomerase; PKC, protein kinase C; ERK, extracellular signal-regulated kinase; GAPDH, glyceraldehyde-3-phosphate dehydrogenase.

Protein	Impact of Glutathionylation	Reference
1-cys Prx (Prdx VI)	restores peroxidase activity	[121,157]
2-cys Prx	restores peroxidase activity	[121,127,128]
NOS	inhibits activity	[129]
PDI	inhibits isomerase activity	[134,136]
Actin	inhibits polymerization	[138,139,140]
Vimentin	inhibits elongation	[8]
Cofilin	reduces depolymerization activity	[8,158]
Myosin	increases Ca^2+^ sensitivity	[8,159]
β-tubulin	inhibits polymerization	[140,160]
p53	reduces DNA binding	[146,147]
PKC	inhibits activity	[153]
Complex-I	inhibits activity	[154]
Cytochrome oxidase	inhibits activity	[8]
adenosine triphosphate (ATP)-ase	inhibits activity	[14]
Carbonic anhydrase	inhibits activity	[155]
Pyruvate dehydrogenase	inhibits activity	[156]
ERK	inhibits activity	[153]
protein-tyrosine phosphatase (PTP1B)	inhibits activity	[14]
Phosphatase and tensin homolog (PTEN)	inhibits activity	[161]
Aldolase	inhibits activity	[140]
Adenylate kinase 2	inhibits activity	[8]
Vimentin	inhibits activity	[8]
c-Jun	inhibits activity	[155]
NF-κB subunits 65 and 50	inhibits activity	[162]
HSP60	inhibits activity	[8]
HSP70	inhibits activity	[8]
S100 A1, S100 A4, S100 B	increases activity	[163]
Nicotinamide adenine dinucleotide hydrogen (NADH) ubiquinone reductase	inhibits activity	[164]
Inhibitor of nuclear factor kappa B kinase (IKK) β-subunit	inhibits activity	[165]
GAPDH	inhibits activity	[166]
Caspase 3	inhibits activity	[155,167]
SerpinA1 and A3	inhibits activity	[168]
TRAF2	inhibits activity	[134]
STAT3	inhibits activity	[169]
Src homology region 2 domain-containing phosphatase 1 and 2 (SHP-1, SHP-2)	inhibits activity	[170]
Thioredoxin (Trx)	inhibits activity	[171]
p12	inhibits activity	[167]
p17	inhibits activity	[167]
Sarco/endoplasmic reticulum Ca2+-ATPase (SERCA)	increases activity	[172]
CCAAT/enhancer-binding homologous protein (CHOP)	inhibits activity	[134]
Protein kinase B (Akt)	increases activity	[62]
Calreticulin	inhibits activity	[166]
Enolase 1 (Eno1)	inhibits activity	[166]
High mobility group box 1 (HMGB1)	inhibits activity	[173]
Ras	increases activity	[174]
